# New Approaches to Radiation Protection

**DOI:** 10.3389/fonc.2014.00381

**Published:** 2015-01-20

**Authors:** Eliot M. Rosen, Regina Day, Vijay K. Singh

**Affiliations:** ^1^Departments of Oncology, Biochemistry and Molecular & Cellular Biology, and Radiation Medicine, Lombardi Comprehensive Cancer Center, Georgetown University School of Medicine, Washington, DC, USA; ^2^Department of Pharmacology, F. Edward Hébert School of Medicine, Uniformed Services University of the Health Sciences, Bethesda, MD, USA; ^3^Department of Radiation Biology, F. Edward Hébert School of Medicine, Uniformed Services University of the Health Sciences, Bethesda, MD, USA; ^4^Radiation Countermeasures Program, Armed Forces Radiobiology Research Institute, Uniformed Services University of the Health Sciences, Bethesda, MD, USA

**Keywords:** radiation protection, radioprotectors, mitigators, cancer treatment, irradiation

## Abstract

Radioprotectors are compounds that protect against radiation injury when given prior to radiation exposure. Mitigators can protect against radiation injury when given after exposure but before symptoms appear. Radioprotectors and mitigators can potentially improve the outcomes of radiotherapy for cancer treatment by allowing higher doses of radiation and/or reduced damage to normal tissues. Such compounds can also potentially counteract the effects of accidental exposure to radiation or deliberate exposure (e.g., nuclear reactor meltdown, dirty bomb, or nuclear bomb explosion); hence they are called radiation countermeasures. Here, we will review the general principles of radiation injury and protection and describe selected examples of radioprotectors/mitigators ranging from small-molecules to proteins to cell-based treatments. We will emphasize agents that are in more advanced stages of development.

## Introduction

Medical countermeasures (MCM) is a term utilized by the Departments of Defense and Health and Human Services that refers to agents used to prevent (protectors and mitigators) or treat (therapeutics) radiation injury. We will not discuss agents that are solely used to treat established radiation injury (therapeutics). Because of the threat of nuclear terrorism or nuclear accidents (e.g., Chernobyl or Fukushima nuclear reactor meltdowns), several governmental agencies [Department of Defense, National Institute of Allergic and Infectious Diseases (NIAID), Biomedical Advanced Research and Development Authority (BARDA), and Defense Advanced Research Projects Agency (DARPA)] have been interested in the development of agents that can protect against the effects of ionizing radiation (IR), increase survival, and/or decrease morbidity. As an additional benefit, some MCMs may be useful as radioprotectors in the radiation therapy clinic provided that they do not equally render tumors more resistant to IR.

## Radiation and Normal Tissue Complications

Most recent advances in radiation oncology related are due to methods to make the radiation beam better conform to the shape of the tumor and thereby reduce the volume of normal tissue within the radiation beam and the dose to normal tissues. These approaches include intensity modulated radiation therapy (IMRT), stereotactic radiosurgery (e.g., using the Gamma Knife or CyberKnife), and proton beam therapy. However, it is not possible to exclude all normal tissues from the radiation field; and normal tissue damage remains a dose-limiting factor in the treatment of some tumor types (e.g., locally advanced cancers of the cervix, lung, head and neck, and brain). Thus, normal tissue radioprotection is a promising strategy to prevent damage to radiosensitive tissues and organs.

Initial studies of radioprotectors and mitigators typically involve investigation of the acute effects of total-body irradiation (TBI) in rodents, using survival as the end-point. While TBI affects multiple organ systems, death in humans and rodents in the first 30 days is mainly due to two mechanisms: (1) gastrointestinal (GI) syndrome, which often leads to death within 10–12 days after exposure to 8–20 Gy of γ-rays, due to fluid and electrolyte imbalance and bacterial translocation (sepsis); and (2) hematopoietic syndrome, which leads to death within 30 days after exposure to 3–8 Gy, due to neutropenia and thrombocytopenia (1–6). The effects of radiation within the first 30 days are called “acute radiation syndrome (ARS)” or “radiation sickness.” ARS follows a similar pattern in humans and rodents, except that the LD_50/30_ values (dose of whole body exposure required to reduce survival to 50% by day 30, without medical support) are lower in humans (ca. 3.5–4 Gy) than in rodents (ca. 7–9 Gy) (7).

An effective radioprotector/mitigator should improve a 30-day survival in rodents by protecting against GI syndrome, hematopoietic syndrome, or both. It should also have a convenient mode of delivery (e.g., by oral, subcutaneous, or intramuscular routes). For hematopoietic syndrome, it is thought that death within the first 30 days is due to depletion of hematopoietic progenitor cells (HPCs) for white blood cell and megakaryocyte lineages, leading to neutropenia and thrombocytopenia (1, 2). HPCs are more radiosensitive than pluripotent stem cells (HSCs) (8–10). However, irradiated HSCs take a long time (30 days or so) to be recruited into the cell cycle and reconstitute neutrophils and platelets. Thus, if an individual survives for 30 days, HSCs will have sufficient time to reconstitute the various bone marrow lineages, and further hematological support is not required.

Gastrointestinal syndrome is due to depletion of intestinal stem cells (ISCs) located at or near the base of the intestinal crypts (11, 12). These cells die rapidly after exposure to a high dose of radiation by apoptosis. PUMA (p53 up-regulated modulator of apoptosis) appears to be a crucial mediator of apoptosis in ISCs. Crypts become progressively denuded as apical cells are shed and ISCs die or enter cell cycle arrest due to DNA damage. The villus length, number of villi per circumference, and mitotic index decrease starting about four days after irradiation (13). Death due to GI syndrome in mice usually occurs within 10-15 days, depending upon the mouse strain and radiation dose. However, in surviving animals (e.g., due to treatment with a radioprotector), crypts begin to regenerate (as indicated by an increase in DNA synthesis) by day 15 or so.

Although the GI system and bone marrow are rapidly reacting systems that contribute to ARS following TBI, high dose partial body radiation that includes the lungs can result in delayed toxicity that occurs 3-10 months after exposure. This syndrome is related to repeated cycles of inflammation, eventually resulting in pulmonary fibrosis and death, depending on the dose and volume of lung irradiated (14–17). The skin and kidneys are also “radiosensitive” tissues in which severe effects can be observed in individuals who receive high dose partial body irradiation. ARS is the best understood consequence of TBI. Less is known about the later effects of high dose partial body irradiation and the late consequences of ARS. Much of what we know about the sensitivity of specific tissues and organs to radiation comes from early experience with radiation therapy, before the radiation tolerances of these tissues and organs were established and before the introduction of skin sparing megavoltage radiation.

Radiation therapy is usually delivered as fractionated treatments using small dose increments (1.8-3 Gy) delivered five days per week to the tumor site(s). Total doses may vary from 30 to 80 Gy, depending upon the intent of treatment (i.e., curative vs. palliative) and the type and location of the tumor. Side effects from radiation have been well-studied and are classified as acute, intermediate, or late effects (18–26). Acute effects occur during a course of radiotherapy and are resolved within 4 weeks after the last treatment. Examples include epidermitis and mucositis due to injury to the skin and mucosal membranes, respectively. Intermediate effects are less common and occur within 8-12 weeks after the end of radiation. An example is radiation pneumonitis, which reflects inflammation of the lung and is typically confined to the radiation portals. Late effects occur at least 9 months after the end of radiation and are usually the dose-limiting factor in clinical radiotherapy. Late effects include injury to specific tissues and organs within the radiation field or in the entrance or exit paths of the radiation beam. Other types of late effects due to irradiation include carcinogenesis (second tumors caused by radiation), teratogenesis (malformation of fetus, which is very rare because pregnant women are rarely treated with radiation), and effects on growth and development due to irradiation in childhood.

The likelihood of a late effect depends on the total dose of radiation, the fraction size, the volume of tissue being treated, and other treatments (e.g., chemotherapy). Late effects also depend upon prior or subsequent surgery, genetic factors unique to the individual patient, pre-existing vascular damage (e.g., diabetes), hypertension, age, and other pre-existing conditions (e.g., inflammatory bowel disease in patients who receive abdominal irradiation). The dose of radiation and/or volume of irradiated tissue is limited due to late effects: e.g., tumors of the brain and spinal cord and locally advanced cancers of the lung, cervix, breast, and head and neck. Here, a selective normal tissue protector could allow a higher dose, a larger treatment volume, and/or reduced late normal tissue injury, thus increasing the therapeutic ratio.

A reduction in early effects (e.g., epidermitis, mucositis, cystitis, and proctitis) due to a radioprotector could increase patient comfort. Although these effects usually resolve by themselves, they sometimes require a treatment break that delays the completion of radiation. Concurrent chemotherapy and radiotherapy can cause severe acute effects (e.g., debilitating mucositis and weight loss); and here a normal tissue protector could be beneficial (27–32). Normal tissue protection could be particularly useful in young children undergoing cranial irradiation by protecting a central nervous system that is not fully developed (33, 34). Effects on the growth of bones (before epiphyseal closure) and the possibility of a second tumor due to radiation must be considered whenever children are treated with radiation alone or in combination which chemotherapy.

A relatively recently recognized late consequence of thoracic and chest wall irradiation (e.g., treatment of Hodgkin’s disease or post-operative radiotherapy for breast cancer) is radiation-induced heart disease (RIHD), which is usually observed at least several years after treatment and is characterized by accelerated atherosclerosis, cardiac fibrosis, valvular damage, and an increased risk of cardiac-related mortality (35, 36). RIHD can occur when part or all of the heart is included in the radiation field. This condition is usually progressive; and its incidence increases with time after treatment. A significantly increased risk of neurovascular events (e.g., stroke or transient ischemic attack) has been observed after cranial irradiation for brain tumors in children (37). Neurocognitive decline after whole brain irradiation in adults (“radiotherapy brain”) is common, particularly in individuals who have also received chemotherapy. Since there is no specific treatment for these complications, a prevention strategy is required.

## Mechanisms of Radiation Injury and Repair

**Figure 1:**
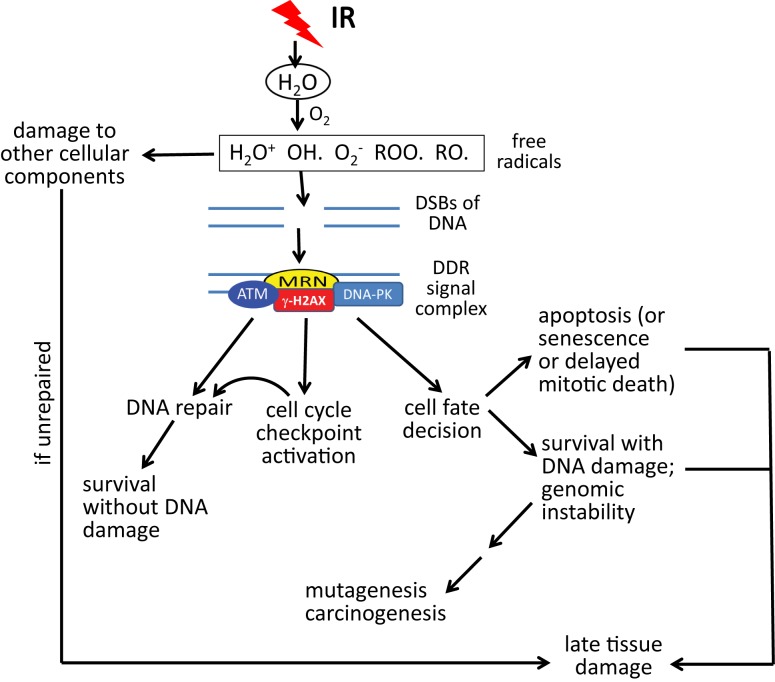
**DNA damage response (DDR) to double-strand DNA breaks (DSBs) in relation to acute radiation syndrome and late effects**. DSBs caused by oxidative radicals are sensed by the MRN complex (MRE11–RAD50–NBS1), resulting in an ATM (ataxia-telangiectasia, mutated)-driven DDR. Gamma-H2AX (phosphorylated histone H2AX protein) is both a participant in the DDR and a marker of DSBs. Depending upon the dose of radiation, the type of radiation, the volume of tissue irradiated, and other factors, the DDR may lead to some combination of DNA repair, permanent cell cycle arrest (senescence), cell death, or survival with DNA damage. As a result of these processes, acute and late radiation effects may ensue, resulting in survival, death, or survival with late tissue damage. Note that “acute radiation syndrome” refers to the consequences of whole body radiation exposure. Acute effects of radiation may be limited to specific tissues or organs in the case of partial body radiation exposures or radiotherapy treatment to tumor-bearing tissue.

Although IR can directly target critical cellular macromolecules such as DNA, water (H_2_O) is by far the most abundant molecule within cells and is thus the most likely target for radiolysis by high energy photons (38–41). As shown in Figure 1, molecular oxygen (O_2_) is a central component involved in the formation of highly reactive free radicals; and so it is not surprising that high concentrations of O_2_ potentiate the effects of IR, while low concentrations of O_2_ (hypoxia) protect cells and tissues from IR, the so-called “oxygen effect” (42–44). The most damaging species of free radical is the hydroxyl radical (OH) (45, 46). DNA is the most critical target for cell survival, but significant damage to other cellular molecules such as proteins and lipids is also produced (47, 48). These oxidative radicals produce two major forms of DNA damage, double-strand breaks (DSBs) (the most lethal form of damage) and base lesions (which are repaired by the base excision repair pathway) (49–52). During the processing of base lesions, single-strand DNA breaks (SSBs) are generated, which are then repaired by one of several mechanisms that involves a scaffolding protein, DNA polymerase, and a DNA ligase. If two base lesions on opposite strands are close enough, the result can be a DSB.

In DSB repair, a DNA-damaging signaling/repair complex accumulates at and around the DSB site. The “MRN” complex of three proteins (MRE11–RAD50–NBS1) senses the damage and binds to the broken DNA ends (53). Following MRN, ataxia telangiectasia mutated (ATM), a nuclear serine/threonine protein kinase, is recruited to the MRN complex and activated through autophosphorylation, after which it phosphorylates a number of substrate proteins on SQ/TQ motifs. The eventual result is the coating of DNA surrounding the break with a set of proteins that orchestrates the DNA repair process. These events are reviewed elsewhere (54). DSB repair can proceed by two pathways: (1) homology-directed repair (HDR) (orchestrated by ATM/BRCA1/BRCA2 signaling), which is an error-free process; and (2) non-homologous end joining (NHEJ), which can be accurate or can lead to significant sequence deletions and translocations [orchestrated by DNA-dependent protein kinase (DNA-PK)] (55, 56). HDR occurs only in S-phase and G2, since it requires a sister chromatid as a template for DNA repair synthesis, while NHEJ can occur in any phase of the cell cycle, but preferably occurs during G1.

In addition to mediating DNA repair, ATM signaling also results in activation of DNA damage-dependent cell cycle checkpoints (e.g., S and G2/M), which allows time for damaged cells to repair their damage, so that it is not passed on to daughter cells (Figure 1). ATM also orchestrates the “cell fate” decision (57). Cells that have too much damage to repair are pushed into rapid death by apoptosis or, alternatively, permanent cell cycle arrest (“senescence”) or delayed death through mitotic catastrophe. ATM can also stimulate cell survival pathways (e.g., the anti-apoptotic transcription factor NF-κB) (58, 59). If cells protected by NF-κB signaling have not fully repaired their DNA damage, this can result in cells with genomic instability, which can result in the accumulation of mutations and, eventually, carcinogenesis, a late effect that usually occurs at a minimum of 3–5 years after radiation exposure (60, 61).

Depending on the dose and proportion of the body exposed to radiation, the relative apoptotic vs. surviving GI and hematopoietic stem/progenitor cell populations may result in ARS (described above), which can lead to death or survival and recovery. In the case of partial body radiation exposure, high dose clinical radiotherapy, or even in survivors of ARS, late complications of radiation may ensue, the seriousness of which depends upon the specific tissue, the radiation dose, and the volume of tissue irradiated. The mechanism(s) of late tissue damage is not fully understood, but may result from damage to parenchymal stem/progenitor cells, blood vessels, inflammation, and/or ongoing oxidative stress due to generation of reactive oxygen species (ROS) (62). Repeated cycles of inflammation may lead to fibrosis [e.g., in lung (62–65)]; and ROS can cause additional DNA damage by causing oxidation of DNA bases, creating a vicious cycle. Possible outcomes include death, survival with permanent late tissue damage of different degrees of severity, or tissue recovery with little or no functional deficit.

## Radioprotectors, Mitigators, and Candidate Agents

### Amifostine as a radioprotector

No radioprotectors or mitigators are currently approved by the Food and Drug Administration (FDA) for general use in humans for the prevention or treatment of ARS. Although amifostine (Ethyol^R^) is not a new agent, to date, it is the only drug that has been approved by the FDA to reduce the toxicity of radiation therapy in the setting of cancer treatment (66). This agent is also used to protect against renal toxicity due to cis-platinum, a DNA cross-linking agent that is also known to cause oxidative stress (67–69). Amifostine (formerly known as “WR-2721”) was originally developed by the U.S. Army Anti-Radiation Drug Development Program as an MCM. It is a thiol compound that acts as a free radical scavenger to reduce the levels of oxidative radicals that would otherwise attack important cellular targets, such as DNA and other cellular macromolecules (70). Amifostine has been used successfully to prevent xerostomia (dry mouth) due to head and neck irradiation, which can otherwise cause permanent dry mouth due to inclusion of the salivary glands (particularly the parotid glands) within the radiation field (71, 72). Initially, there were some concerns that the widespread usage of amifostine would also protect the tumor against radiation or chemotherapy drugs, but accumulated experience has shown that this is not the case (73).

In a recent report that examined 30 studies utilizing amifostine, no conclusion could be made regarding the efficacy of amifostine in preventing or reducing oral mucositis, because of conflicting and confusing data (74). In a recent meta-analysis that included multiple clinical trials in which amifostine was used to prevent cis-platinum toxicity, there was a trend toward a reduction in the incidence of platinum-induced ototoxicity (hearing loss due to cochlear damage), but the trend did not reach statistical significance (75). In a study of locally advanced non-small cell lung cancers treated with chemoradiotherapy plus or minus amifostine, amifostine conferred a significant reduction in pain and dysphagia (difficulty swallowing). And in a study of patients who received postmastectomy radiation without or with amifostine (at different dose levels), patients who received amifostine had a lower incidence of skin toxicity, and pulmonary and soft tissue fibrosis (76). In a recent meta-analysis of cancer treatment trials that tested amifostine to reduce acute side effects, it was concluded that amifostine did not reduce overall survival or progression-free survival in patients who received radiotherapy plus or chemoradiotherapy plus amifostine (73).

The most commonly accepted explanation for the lack of radioprotection of tumors is that amifostine itself (WR-2721) is an inactive pro-drug, which must be converted to an active drug (WR-1065) by dephosphorylation. The conversion is usually due to alkaline phosphatase in the cell membrane of normal endothelium. Tumors, which have abnormal vasculature which is sparser than in normal tissues and contains lower levels of alkaline phosphatase, are much less efficient at activating amifostine than normal tissue [reviewed in Ref. (66)]. Several other mechanisms were proposed to explain the selective radioprotection by amifostine, including protection of DNA by metabolites of amifostine, causing hypoxia in normal tissues by increasing oxygen consumption, and accelerated recovery of normal endothelial cells [reviewed in Ref. (66)].

Amifostine has several clinically relevant limitations including: (1) the need to administer it within a narrow time window (15–30 min) before each radiation dose; (2) its approval only for intravenous use, although other routes of administration (e.g., subcutaneous) are under investigation (76, 77); and (3) toxicity, including nausea, vomiting, somnolence, and hypotension. Recently, it has been demonstrated that radioprotective doses for amifostine appear to lie between 25 and 50 mg/kg in mice. Mature, lineage-restricted progenitors appear to be more responsive to the protective effects of low doses of amifostine than the more primitive, multipotential progenitors (78).

### Palifermin

Palifermin is a recombinant N-terminal truncated form of keratinocyte growth factor [KGF, also known as fibroblast growth factor 7 (FGF7)], a growth factor that is produced by mesenchymal cells and acts in a paracrine manner to stimulate the proliferation of epithelial cells. KGF generally functions in the protection and repair of epithelial tissues through its cognate receptor FGFR2B. Its protective action appears to be due to a combination of stimulation of cell proliferation and protection against apoptosis (79). Oral mucositis is a significant toxicity in patients undergoing radiotherapy and particularly chemoradiotherapy for head and neck cancers. This complication frequently requires a treatment break and reduces the quality of life in patients receiving such treatment. Here, severe oral mucositis can result in weight loss due to reduced oral intake, requirement for pain medicines, increased risk of infections, and, in some cases, the need for hospitalization. Mucositis is due, in part, to an imbalance between death and shedding of oral mucosal lining cells and the ability of cells newly recruited into the cell cycle to replace the lost cells. The result is a partial denudation of the mucosal surface. Oral mucositis has been traditionally managed by supportive care, including basic oral care hygiene, appropriate pain management, and the use of mouthwashes and oral rinses.

A variety of preclinical studies suggested that palifermin could ameliorate the mucosal toxicity due to chemotherapy and/or radiation therapy (79). Palifermin was first approved by the FDA in 2004 for the indication of preventing severe oral mucositis in patients undergoing hematopoietic stem cell transplantation for the treatment of hematological cancers (80, 81). These patients receive high dose chemotherapy without or with total-body radiotherapy prior to transplantation and are at-risk for severe (grades 3–4) mucositis. Beneficial effects of palifermin were documented in patients receiving palifermin for 3 days prior to the preparative regimen and for 3–5 days after transplantation. The use of palifermin has also been shown to reduce the incidence, duration, and severity of oral mucositis in patients treated with chemoradiotherapy for head and neck cancers and in patients receiving chemotherapy using agents that can cause mucositis, including adriamycin and 5-fluorouracil. Palifermin has also been utilized to mitigate dysphagia due to esophagitis in patients treated with chemoradiotherapy for lung carcinoma. Interestingly, in addition to mucositis, palifermin also appears to stimulate immune reconstitution following hematopoietic stem cell transplantation and to reduce graft-vs.-host disease following allogeneic bone marrow transplantation (80). Palifermin is generally well-tolerated but its usage has been associated with skin rash and taste disturbance.

### Superoxide dismutase

Greenberger and colleagues have been studying the use of a superoxide dismutase (SOD) transgene to protect normal tissues against injury due to IR. Here, over-expression of manganese superoxide dismutase (MnSOD, also called SOD2) by intra-tracheal injection of a replication deficient adenovirus containing the MnSOD transgene conferred protection against lung irradiation and cytokine production (IL-1, TNF-alpha, and TGF-beta) when administered prior to irradiation (82). Interestingly, intra-tracheal administration of the MnSOD transgene protected normal lung but not orthotopic Lewis lung carcinoma against pulmonary irradiation (83). Similarly intraesophageal administration of MnSOD prevented the development of radiation-induced esophagitis and modulated cytokine expression (84, 85). In both the lung and esophageal models, the MnSOD transgene was well-expressed in the respective normal tissues. Incultured cell lines, the MnSOD appeared to work, in part, by protection against radiation-induced apoptosis via stabilization of the mitochondrial membrane (86). In a mouse model of radiation-induced oral mucositis, a significant form of radiation-induced injury in patients receiving head and neck irradiation, intraoral administration of MnSOD caused a decrease in the extent of radiation-induced ulceration (87). As in the case of lung and esophageal irradiation, MnSOD did not confer protection of head and neck carcinoma (87). Here, addition of amifostine to the MDSOD did not confer additional protection beyond that due to MnSOD alone. As Cu/ZnSOD (SOD1) did not protect mice against thoracic irradiation, it appears that mitochondrial localization and prevention of mitochondria-induced apoptosis figure into the mechanism of radiation protection by MnSOD (88).

Together, these findings suggest the possibility of utilizing radioprotective antioxidant gene therapy to prevent or reduce the extent of some forms of radiation injury. Here, it also appears that transgene expression in cells within the microenvironments of protected organs contribute significantly to the protection (89). In this regard, a phase I study of MnSOD administered orally was carried out in patients who received a standard chemoradiation regimen for for stage III unresectable lung carcinoma. In this study, there did not appear to be any dose-limiting additional toxicity due to administration of the MnSOD transgene at any of three doses (up to 30 mg per patient). Interestingly, Greenberger and colleagues also showed that in bone marrow stromal stem cells, doxycycline induced expression of a tetracycline regulated MnSOD conferred radiation resistance, whereas in the absence of doxycycline, the cells showed normal radiation sensitivity (90). Mice fed a diet rich in antioxidants and given MnSOD showed an increased lifespan as compared with MnSOD plus house diet, following TBI to 9.5 Gy (91). These findings suggest that when combined with MnSOD, the antioxidant/chemopreventive diet reduces the extent of radiation-induced life-shortening due to TBI in survivors of the ARS. There was no increase in detectable tumors, or histopathologic evidence of neurodegenerative disease in the increased number of survivors following MnSOD plus irradiation. Intravenous administration of MnSOD also ameliorated the growth retardation in the newborn mice from irradiated mothers (92). And MnSOD when given 24 h prior to irradiation of pregnant mice conferred a significantly increased number of live births. The protection appeared to be due to a remote (bystander) effect, since increased expression of MnSOD in fetal tissues could not be demonstrated by RT-PCR in this study.

### Other radioprotective agents

Interestingly, in a screen of 13 drugs utilized during bone marrow transplantation, tetracycline, but not other antibiotics and anti-fungal agents appeared to protect cultured HPCs against IR (93). As protection was observed in radiation dose–response assays, these findings suggest that the observed radioprotection of tetracycline is not due directly to its properties as an anti-microbial agent. p53 Up-regulated modulator of apoptosis (PUMA) is a Bcl-2 homology 3 (BH3)-only Bcl-2 family member that has been implicated in radiation-induced apoptosis. Recently, a group of pharmacophiles that inhibit PUMA and radiation-induced apoptosis have been identified (94, 95). These agents appear to work, in part, by disrupting the interaction between PUMA and Bcl-X_L_. In other studies, a p53/Mdm2/Mdm4 inhibitor, BEB55, protected mice against radiation-induced esophagitis when administered orally (96).

### Genistein

Genistein (4′,5,7-trihydroxyisoflavone) is a soy isoflavone with a variety of cellular activities, including selective estrogen receptor activation, protein tyrosine kinase inhibition, antioxidant activity, and free radical scavenging activity (97–100). Genistein has been established as an anti-cancer agent, and has additionally been demonstrated to have anti-microbial and anti-inflammatory activity *in vivo* (101–105). Genistein was reported in clinical trials to reduce the adverse effects of chemotherapy and radiotherapy (106, 107). The protective effects of genistein for radiation-induced injury to the bone marrow were observed in a murine model of ARS, where neutrophils and platelets were protected (108, 109). Genistein also protected bone marrow progenitor cell populations, thus preventing hematopoietic stem cell pool exhaustion (109, 110). Genistein administration reduced radiation-induced injury in the lung and increase survival from thoracic irradiation in mice (111). Genistein reduced micronuclei in Lin^−^ bone marrow cells and primary lung fibroblasts suggesting a direct reduction of radiation-induced DNA damage (17, 111–113). Several mechanisms have been proposed for radioprotective effects by genistein, including activation of the DNA repair enzyme Gadd45 (114–116), the quiescence of the cell cycle of Lin^−^ cells in the G_0_/G_1_ phase *in vivo* (110, 117), and the suppression of inflammation (14, 105, 118, 119).

### Captopril and ACE inhibitors

Captopril, a sulfhydryl-containing analog of proline, is a competitive inhibitor of the angiotensin converting enzyme (ACE) protease, and reduces systemic blood pressure by blocking both the activation of the vasoconstrictor angiotensin II (Ang II) and the inactivation of the vasodilator bradykinin. Although captopril was initially developed for the treatment of hypertension and heart failure, it was found that captopril was also useful in animal models of radiation-induced renal dysfunction for increasing renal plasma flow and improving glomerular filtration (120, 121). Captopril has been investigated as a radiation countermeasure for the pulmonary, renal, and hematopoietic systems as well as for the brain and skin (122–127). ACE inhibitors and captopril mitigated radiation-induced pulmonary endothelial dysfunction, radiation pneumonitis, and fibrosis in animal models (128, 129). Prophylactic administration of captopril resulted in lower systemic blood pressure and improved renal function following TBI in animal models (121, 130, 131) and reduced chronic renal failure in human patients undergoing clinical radiation (132). Captopril and another ACE inhibitor, perindopril, were demonstrated to block radiation-induced hematopoietic syndrome through accelerated recovery of erythrocytes, reticulocytes, leukocytes, and platelets (122, 133). The improved blood cell recovery was associated with improved survival of specific hematopoietic progenitor populations CFU-GM, CFU-M, and total CFC (122). The mechanism of captopril-induced reduction of radiation injury has not been established. Captopril mitigation of radiation injuries may involve reduced inflammation (134) or the transient quiescence of some cells *in vivo* (122, 135). However, *in vivo* effects on radiation-induced DNA damage have not been shown (112).

### 3,3′-Diindolylmethane

3,3′-Diindolylmethane is a small-molecule compound formed by acid hydrolysis in the stomach of indole-3-carbinol (I3C), a component of cruciferous vegetables (e.g., cabbage, cauliflower, and broccoli) (136). 3,3′-Diindolylmethane (DIM) is a proposed cancer prevention agent that is available as a nutritional supplement and has been administered safely by the oral route to humans in repeated doses in phase I/II clinical trials (137–140). Recently, it was found that administration of DIM in a multidose schedule protected rodents against lethal doses of TBI up to 13 Gy, whether DIM dosing was initiated 24 h before or up to 24 h after irradiation (141). The dose reduction factor (DRF) (i.e., ratio of LD_50/30_ values in the presence/absence of DIM) was 1.6 when DIM treatment was begun 24 h after irradiation. Low physiologically relevant (submicromolar) concentrations of DIM protected cultured cells against radiation by a novel mechanism. DIM caused rapid activation of ATM and phosphorylation of various ATM substrates, suggesting that DIM induces an ATM-dependent DNA damage response (DDR)-like response, and DIM enhanced radiation-induced ATM signaling and NF-κB activation. Similarly, DIM caused ATM activation and signaling in normal tissues in rodents. However, DIM did not protect human breast cancer xenografts (MDA-MB-231) against radiation. In the tumors, ATM signaling appeared to be defective. The results appear promising, but further work is required to determine if DIM will be a useful radioprotector and/or mitigator.

3,3′-Diindolylmethane was also shown to have cardioprotective properties. Here, subcutaneous administration of DIM decreased the extent of fibrosis due to adriamycin, a DNA-damaging chemotherapy agent by a mechanisms that involves up-regulation of BRCA1 and activation of the antioxidant transcription factor nuclear factor (erythroid-derived 2)-like 2 (NFE2L2) (142). DIM mediated cardioprotection against other stressors including aortic banding, which causes cardiac hypertrophy, due to a mechanism involving 5′-adenosine monophosphate-activated protein kinase-alpha2 (AMPK-α2) and mammalian target of rapamycin (mTOR) (143). Whether DIM will protect the heart against IR has not been described.

### Inhibitors of radiation-induced accelerated senescence

Loss of cellular clonogenic potential following exposure to radiation can be caused by apoptosis, necrosis, autophagy, and accelerated cellular senescence. Recent findings suggest that accelerated cellular senescence may be a primary effect of radiation on normal (non-transformed, non-immortalized) epithelial and endothelial cells and fibroblasts. Cellular senescence results in a range of aberrant biological activities and can influence overall tissue dysfunction (144–150). The blockade of radiation-induced cellular senescence by the pharmacological inhibitors of mTOR was sufficient to prevent mucositis in mice following irradiation of the head and neck area (145). In this study, it was demonstrated that rapamycin blocked radiation-induced senescence, but not apoptosis, in primary keratinocyte in cell cultures and *in vivo* in a murine model of head/neck irradiation injury.

Investigation into receptor signaling pathways that contribute to aging-associated cellular senescence revealed the involvement of the insulin-like growth factor-1 receptor (IGF-1R) (151, 152). IGF-1 enhances senescence in primary cell cultures via a mechanism that involves increase in ROS leading to induction of the p53/p21 pathway (153). In mouse embryonic fibroblasts, treatment with IGF-1 inhibits the deacetylase activity of Sirtuin 1 (SIRT1) and promotes stability of p53, ultimately leading to induction of senescence (154). IGF-1R expression levels increase during the development of replicative *in vitro* senescence in primary cortical neurons (155). In agreement with these findings, a recent study demonstrated that inhibition of IGF-1R, PI3K, and mTor blocked radiation-induced accelerated senescence in primary lung endothelial cells in cell culture (156).

### CBLB502/Entolimod™

CBLB502 is a potent and stable agent derived from the flagellin protein of *Salmonella* bacteria (*Salmonella enterica serovar Dublin*). Its pharmacologic action is based on binding to toll-like receptor 5 (TLR5) of targeted cells and activating NF-κB signaling. Biologically, purified flagellin protects mice from lethal doses of total-body gamma-irradiation (157). Cleveland BioLabs, Inc. (Buffalo, NY, USA) identified CBLB502 (now known as Entolimod) as a TLR5 ligand that significantly improved the radioprotective efficacy of flagellin while having reduced toxicity and immunogenicity (158).

A single injection of CBLB502 either before lethal TBI (24 h prior) or up to 48 h following irradiation protected mice from both GI and hematopoietic syndromes, with significantly improved survival. CBLB502 also demonstrated radioprotective and radiomitigative potential in lethally irradiated non-human primates (158). A single intramuscular injection of CBLB502 significantly increased the survival of rhesus non-human primates exposed to 6.5 Gy TBI and promoted the regeneration of their small intestine, spleen, thymus, and bone marrow when administered from 1 to 48 h after irradiation. The severity and duration of irradiation-induced thrombocytopenia and neutropenia decreased significantly with CBLB502 treatment. Two cytokines, granulocyte colony-stimulating factor (G-CSF) and interleukin-6 (IL-6) were identified as candidate biomarkers for the radioprotective and radiomitigative efficacy of CBLB502. Induction of both G-CSF and IL-6 by CBLB502 is TLR5-dependent, dose-dependent within its efficacious dose range in both unirradiated and irradiated mammals (including rodents and non-human primates), and critically important for the CBLB502-mediated increased survival of irradiated animals (159). Administration of either G-CSF or IL-6 neutralizing antibody abrogated the radiomitigation by CBLB502. These biomarkers are likely to be useful for the accurate prediction of CBLB502 dose providing radioprotection or radiomitigation in humans. Furthermore, CBLB502 was shown to significantly reduce the severity of dermatitis and oral mucositis caused by local radiation exposure (160). The FDA has granted IND status to CBLB502 as a radiation countermeasure for ARS and it is currently in clinical development.

### ON01210/Ex-RAD®

ON01210 (a chlorobenzylsulfone derivative known as Ex-RAD) is a novel, small-molecule kinase inhibitor under development as a radiation countermeasure. Ex-RAD provided significant protection against cobalt-60 gamma-irradiation when administered sc (500 mg/kg) to C3H/HeN mice 24 h and 15 min before irradiation. Ex-RAD’s estimated DRF is 1.16 (161). In another study, Ex-RAD showed a significant survival benefit after prophylactic oral administration of the drug (162).

This drug accelerated the recovery of peripheral blood elements in irradiated mice when administered either subcutaneously (sc) or orally (162, 163). In addition, Ex-RAD-treated mice (either through the oral or sc route) contained higher numbers of granulocyte macrophage-colony forming units (GM-CFUs) than in vehicle-treated mice. Bone marrow obtained from irradiated mice indicated that Ex-RAD protected cells from radiation-induced apoptosis after exposure to cobalt-60 gamma-irradiation (163). Ex-RAD also assists in the recovery of the GI system, with a higher number of surviving intestinal crypts after acute radiation exposure in Ex-RAD-treated mice than untreated irradiated controls (163). These effects may be due in part to signaling pathways that are affected by Ex-RAD. Attenuation of ATM-p53 mediated DDR by Ex-RAD contributes to the mitigation of radiation-induced hematopoietic toxicity (164). Recently, Kang et al. demonstrated that Ex-RAD manifests its protective effects through the up-regulation of phosphatidylinositol-3-kinase/AKT pathways in cells exposed to radiation (165). Ex-RAD has been granted FDA IND status and has demonstrated oral efficacy (162). Oral administration holds better clinical promise as an effective countermeasure for first responder use as well as for at-risk civilian populations in a nuclear accident.

### Gamma-tocotrienol

Gamma-tocotrienol is one of the eight isomers (tocols) of vitamin E. It is a potent inhibitor of HMG-CoA (3-hydroxy-3-methylglutaryl-coenzyme A) reductase. Gamma-tocotrienol (GT3) has been shown to increase survival in rodents, through ameliorating the hematopoietic and GI systems (166). When administered 24 h before cobalt-60 gamma-irradiation, GT3 significantly protected mice against radiation doses as high as 11.5 Gy, and its DRF as a radioprotector (24 h before irradiation, 200 mg/kg dose, sc route) was 1.29 in mice. GT3 treatment accelerated hematopoietic recovery in peripheral blood and enhanced recovery of hematopoietic progenitors in bone marrow of irradiated mice (167, 168). GT3 treatment resulted in significant induction of G-CSF and IL-6 in mice (170). Mouse survival studies with GT3 suggested the most efficacious time for drug administration was 24 h prior to irradiation, possibly due to the induction of key hematopoietic cytokines during that time window. Prophylactic GT3 administration caused up-regulation of anti-apoptotic genes and down regulation of pro-apoptotic genes (both at the transcriptional and the protein levels) at 4 and 24 h after irradiation (169). The administration of G-CSF antibody abrogated the radioprotective efficacy of GT3 (170).

### δ-Tocotrienol

δ-Tocotrienol has demonstrated antioxidant activity greater than that of γ- and α-tocotrienol in the membrane system while protecting primary neuronal cells against glutamate toxicity (166). A single sc injection of δ-tocotrienol before or after cobalt-60 γ-irradiation significantly protected mice in a 30-day survival experiment. δ-Tocotrienol was effective at a wide dose range of 19–400 mg/kg (171, 172). The DRF values for radioprotective treatment (24 h before irradiation) with 150 and 300 mg/kg were 1.19 and 1.27, respectively. For radiomitigation treatment with 150 mg/kg of δ-tocotrienol administered 2 h after irradiation, the DRF was 1.1. When δ-tocotrienol was administered at 300 mg/kg dose 24 h before irradiation, it significantly reduced radiation-induced cytopenia, suggesting its stimulatory effects on hematopoietic recovery (171). Similar to countermeasures mentioned above, we have demonstrated that the administration of G-CSF antibody abrogates the radioprotective efficacy of δ-tocotrienol (173, 174). Recently, it was demonstrated that δ-tocotrienol reduces activation of caspases 3, 7, and 8 while increasing autophagy-related beclin-1 expression in irradiated bone marrow cells (175). δ-Tocotrienol has been reported to increase cell survival and regeneration of hematopoietic microfoci and lineage^−^/Sca-1^+^/c-Kit^+^ stem and progenitor cells in irradiated mouse bone marrow cells. δ-Tocotrienol also protected CD34^+^ cells from radiation-induced damage (172).

### R-spondin1

Human R-spondin1 (Rspo1), a 29 kDa, 263 amino acid protein acts as a mitogenic factor for ISCs and it was hypothesized that its systemic administration would amplify intestinal crypt cells, accelerate regeneration of irradiated intestine and ameliorate radiation-induced GI syndrome. Mice receiving recombinant adenovirus expressing human R-spondin1 (a potent Wnt signal enhancer and one of the four analogs of R-spondin) before potentially lethal TBI or local abdominal irradiation had higher survival than the control group (176). Rspo1 promoted radioprotection against radiation-induced GI syndrome and improved survival of mice. The mechanism was likely related to induction of the Wnt/β-catenin pathway and promotion of ISC regeneration. Rspo1 has a protective effect only on normal intestinal tissue but not in tumors and thereby may increase the therapeutic ratio of chemoradiation therapy in patients undergoing abdominal irradiation for GI malignancies.

### Transforming growth factor-β3

Radiation-induced pulmonary fibrosis is a frequently occurring complication from radiotherapy of thoracic tumors. The transforming growth factor-β superfamily plays a key regulatory role in pulmonary fibrosis. A single thoracic irradiation of 20 Gy was applied in mice to establish the model of radiation-induced pulmonary fibrosis and the mice were treated by intraperitoneal injections of recombinant transforming growth factor-β3 weekly after irradiation (177). Transforming growth factor-β3 decelerated the progress of radiation-induced pulmonary fibrosis and hindered the recruitment of fibrocytes to lung. In addition, Th1 response was suppressed as shown by diminished interferon-γ in transforming growth factor-β3 after irradiation, and enhancement of Th2 response was marked by increased interleukin in transforming growth factor-β3. These data suggest that TGF-β3 might be involved in the regulatory mechanism for attenuation of radiation-induced pulmonary fibrosis.

## Injury-Mitigating, Therapeutic Cell Transplants: Cellular Therapy

### Mesenchymal stem cells

There has been an explosion of interest in adult stem/progenitor cells that have the potential to repair tissues to treat individuals for a broad range of clinical indications (178). These cells attracted attention because of their stem-cell-like properties, but the cells frequently repair injured tissues without much evidence of either engraftment or differentiation. These cells have been shown to secrete a large numbers of cytokines and chemokines (179, 180). The pattern of secreted cytokines changes after the cell engraftment into new microenvironments suggesting that these cells could enhance repair by stimulating the regeneration of damaged cells. These cells also suppressed the mixed-lymphocyte reaction in culture indicating tissue repair by suppressing immune reaction.

Mesenchymal stem cells have been reported to repair various tissues damaged by radiation exposure when injected intravenously (180). As stated above, the stemness of these cells was probably not relevant to their efficacy in such indications, and it may even be a drawback when possible complications associated with the use of such cells are considered (181, 182). In such cases, cells with low antigenicity and with minimal differentiation potential but with adequate secretion of key modulators of inflammation and immunity such as prostaglandin E2, tumor necrosis factor-stimulated gene 6, and stanniocalcin-1 may profile more optimal candidates. Furthermore, intravenous administration of mesenchymal stem cells (MSCs) genetically modified with extracellular superoxide dismutase improved survival in irradiated mice (183).

### Bone marrow stromal cells

There is a report suggesting that mitigation of lethal intestinal injury can be achieved by intravenous transplantation of marrow-derived stromal cells (including mesenchymal, endothelial, and macrophage cell population) (184). Bone marrow-derived adherent stromal cell transplantation increased blood levels of intestinal growth factors (R-Spondin1, keratinocyte growth factor, platelet-derived growth factor, fibroblast growth factor-2, and anti-inflammatory cytokines) and induced regeneration of the irradiated host ISCs niche. These findings provided a platform to discover potential radiation mitigators and protectors for ARS and chemoradiation therapy of abdominal malignancies.

### Myeloid progenitor cells

Cellerant Therapeutics (San Carlos, CA, USA) has developed culture conditions to produce large numbers of mouse myeloid progenitors from hematopoietic stem cells. Myeloid progenitor cells (MPCs) can improve survival against high levels of radiation. In collaboration with Cellerant Therapeutics, one of us (VKS) studied MPCs for use as a bridging therapy for radiation injuries (185). The aim of this study was to elucidate the potential of mouse myeloid progenitor cells (mMPC) to mitigate lethal doses of ^60^Co γ-radiation and X-rays in various strains of mice. Different cell-doses of pooled allogeneic mMPC generated *ex vivo* from AKR, C57Bl/6, and FVB mice were transfused iv into haplotype-mismatched recipient BALB/c or CD2F1 mice at various times after irradiation to assess their effect on a 30-day survival. Our results demonstrated that cryopreserved allogeneic mMPC significantly improve survival in both strains of mice irradiated with lethal doses of ^60^Co γ-radiation (CD2F1, 9.2 Gy) and X-ray exposures (BALB/c, 9 Gy) that are known to cause ARS in hematopoietic tissues (185). The survival benefit was mMPC-dose-dependent and significant even when mMPC administration was delayed up to 7 days post-irradiation. It was further shown that mMPC administration mitigates death from ARS at radiation doses up to 15 Gy (^60^Co γ-radiation, CD2F1 mice), which are radiation exposure levels that cause mice to succumb to multi-organ failure, and determined that the DRF of 5 million mMPC administered 24 h post-irradiation of CD2F1 mice is 1.73. Even at high doses of up to 14 Gy cobalt-60 gamma-radiation, mMPC administration could be delayed up to 5 days in CD2F1 mice and still provide significant benefit to a 30-day survival. Additional study is needed to monitor mMPC transplanted mice for long term to investigate graft vs. host disease, and to evaluate the histopathology of various organs of transplanted mice. To study the GI tract structural integrity in mice receiving higher doses of radiation exposure causing GI injury and mMPC treatment, intestinal tissues were harvested at different times after irradiation and analyzed for architecture, surviving crypts, villus height, and number. The effect of infused mMPC on bacterial translocation from gut to heart, spleen, and liver in irradiated mice was studied by bacterial tissue cultures and estimated endotoxin levels in serum samples. It was observed that the infusion of mMPC significantly improved survival of mice receiving high doses of radiation, decreased bacterial infection, and lowered endotoxin levels in serum. The histopathology of jejunum from irradiated and mMPC-transfused mice revealed improved gut structural integrity compared to untreated controls. In brief, the results of this study further support our contention that the transfusion of mMPC acts as a bridging therapy, not only for the hematopoietic system, but also for GI system recovery following acute, potentially lethal radiation injury by improving intestinal structural integrity and inhibiting bacterial translocation in the GI tract of lethally irradiated mice.

### Mobilized blood hematopoietic stem cells and early progenitors: tocopherol succinate-mobilized progenitor cells

It was hypothesized that **tocopherol succinate (TS)** would stimulate a G-CSF-induced mobilization of bone marrow progenitor cells into the peripheral circulation. This hypothesis was confirmed clearly using several different approaches (186). First, a direct fluorescence flow cytometric approach was used to identify and phenotype the putative, mobilized hematopoietic stem cells in question (186). Second, we evaluated and compared the efficacy of whole blood infusions obtained from TS-treated mice vs. G-CSF-treated mice for survival protection against hematopoietic ARS when transfused into matched groups of acutely irradiated recipient mice. Survival was significantly higher in the group receiving transfused blood from TS-treated animals (187). Further, our results demonstrated that infusions of HSC-enriched, peripheral blood mononuclear cells (PBMC) from TS-injected mice greatly improved survival of lethally irradiated mice (187). Once transfused, these *TS-mobilized progenitors* acted as a *bridging therapy* for acutely irradiated, morbidly injured mice and the fostering of time-critical recovery process(es) that principally involve damaged cell-replacement and tissue renewal that aid-and-abet overall restoration of vital organ-system function(s).

Recent studies have yielded a remarkable finding; namely, infusion of whole blood or PBMC from TS- and AMD3100-injected mice significantly improved survival of mice receiving still higher, GI-syndrome-eliciting radiation doses. Histopathology and immunostaining of jejunum from these irradiated and TS- and AMD3100-mobilized PBMC-transfused mice revealed significant protection of GI tissue from radiation injury (188). We also observed that the infusion of PBMC from TS- and AMD3100-injected mice significantly inhibited apoptosis, increased cell proliferation in the analyzed tissues of recipient mice, and inhibited bacterial translocation to various organs compared to mice receiving cells from vehicle-mobilized cells (189). Most recently, we have observed that TS-mobilized progenitors mitigate radiation combined injury (radiation and wound) (190). In aggregate, these rodent-based studies strongly suggest that TS has the capacity to mobilize progenitors from marrow into the blood. This subset of unique, tissue-reparative progenitors not only is therapeutic for a critically injured/failing lymphohematopoietic system but also for the GI system and perhaps other vital organ systems as well. Together these characteristics make TS-mobilized progenitors a suitable candidate as a bridging therapy for acute radiation victims that can be administered in the field with minimal infrastructure requirements.

## Conclusion and Perspectives

There are several promising radiation countermeasures under development such as CBLB613 (191), CBLB612 (192), IL-12 (193), epidermal growth factor (194), fibroblast growth factor-2 (195), fibroblast growth factor-peptide (196), insulin-like growth factor-1 (197), tempol (198), TS (13), TPO (thrombopoietin) receptor agonist (ALXN4100TPO) (199), 5-Androstenediol (5-AED)/Neumune^®^ (200), AEOL-10150 (201), cytokines, and growth factors (3, 166, 202), etc. Since it was not possible to discuss all agents under development in this review, we selected some of those agents, which are at advanced stages of the development or are otherwise representative of three general categories of agents: small-molecules, proteins, and cellular therapy. The characteristics of the agents described herein are summarized in Table 1.

**Table 1:** **Summary of radioprotector/mitigator agents and their characteristics**.

Agent name	Agent type	Target tissue(s)	Mechanism(s) of action
Amifostine	Small-molecule (thiol)	Salivary glands mucosa	Free radical scavenger and other (see text)
Tetracycline	Small-molecule (antibiotic)	Bone marrow (HPC protector)	Unknown mechanism not related to its anti-microbial properties
Genistein	Small-molecule (soy isoflavone)	Bone marrow (HPC protector)	Multiple mechanisms (e.g., anti-inflammatory, antioxidant, free radical scanger, stimulator of DNA synthesis)
Captopril (also perindopril)	Small-molecule (anti-hypertensive drug)	Kidney protector, lung, bone marrow (HPC protector)	Angiotensin onverting enzyme (ACE) inhibitor, reduced inflammation; mechanism unclear
3,3′-Diindolylmethane (DIM)	Small-molecule (indole derivative)	GI system bone marrow	Simulates ATM signaling and DNA damage response protection against oxidative stress
Rapamycin	Small-molecule (MTOR inhibitor)	Head and neck mucosa	MTOR inhibitor; blocks radiation-induced cellular senescence
ON01210/Ex-Rad	Small-molecule (chlorobenzyl sulfone derivative)	Bone marrow GI system	Tyrosine kinase inhibitor; attenuation of ATM/p53 signaling; up-regulation of PI3 kinase signaling
γ-Tocotrienol (GT3)	Small-molecule (vitamin E isomer)	Bone marrow (HPC protector) GI system	Antioxidant
δ-Tocotrienol	Small-molecule (vitamin E isomer)	Bone marrow	Antioxidant
Palliformin	Protein (keratinocyte growth factor)	Oral and esophageal mucosa	Stimulates epithelial cell proliferation; inhibits apoptosis
Superoxide dismutase (MnSOD)	Protein (enzyme, delivered by gene therapy approach)	Lung, esophagus, oral mucosa	Metabolizes ROS
CBLB502 (entolimod)	Protein (flagellin derivative)	GI system, bone marrow, skin, oral mucosa	TLR5 agonist; stimulates NF-κB signaling, induction of protective cytokines
TGF-β3	Protein (transforming growth factor-β3)	Lung	Attenuates radiation-induced pulmonary function
R-spondin1 (Rspo1)	Protein (intestinal cell mitogen)	GI system	Accelerates regeneration of irradiated intestine through the Wnt/β-catenin pathway
Mesenchymal stem cell (MSC) transplant	Cellular therapy	Bone marrow various other tissues	Engraft and differentiation of MSCs; cytokine production; suppression of immune response and inflammation
Myeloid progenitor cells (MPCs)	Cellular therapy	Bone marrow GI system	HPC reconstitution; preserves structural integrity of the gut
Tocopherol succinate (TS)-mobilized progenitor cells	Cellular therapy	Bone marrow GI system	HPC reconstitution, bridging therapy; protection of the GI system
Bone marrow stromal cells	Cellular therapy	GI system	Increases blood levels of intestinal growth factors; induces regeneration of intestinal stem cells

We have reviewed some of the principles of radiation protection and mitigation and discussed some of the agents under development. The agents described represent various molecule types, including gene therapy, small-molecule drugs, and drug-like compounds (e.g., captopril, Ex-RAD), phytochemicals (plant-derived agents) (e.g., DIM, genistein), vitamins (e.g., vitamin E derivatives: gamma and delta tocotrienol), protein (e.g., truncated flagellin, CBLB502), and cell-based agents. The wide variety of agents that can function as protectors or mitigators is consistent with the complexity of the responses of different cell types and tissues to radiation. Examples of agents that protect normal tissues but not tumors have been provided. Some such agents may exhibit antitumor activity, particularly at higher concentrations, for example DIM and genistein, which are proposed cancer prevention agents. Many questions remain, such as why some compounds are strong protectants but weak mitigators (e.g., vitamin E derivatives) and why protectants often selectively target normal tissues and not tumors.

Compounds being considered as radioprotectors and/or mitigators are typically tested in rodents using a 30-day survival as the major end-point and in non-human primates (monkeys) using a 60-day survival end-point. These time intervals were chosen to reflect the ability of the compound to protect against or mitigate ARS following whole body exposure to nuclear radiation (e.g., ^60^Co or ^137^Cs). Later effects of whole body, near whole body, or partial body exposures, including survivors of ARS are a relatively understudied area in the field. These may be important because there are other radiosensitive tissues than bone marrow and intestine, including skin, esophagus, lung, and kidney. The performance of radioprotectors/mitigators in the setting of exposure to particulate radiation – for example – neutrons, protons, and heavier ions – is another unexplored area, as most studies utilize gamma-radiation or X-rays. It is also of interest whether a potential radioprotector/mitigator can block radiation-induced mutagenesis and, thus, carcinogenesis, since certain medical procedures (e.g., computerized axial tomography) are associated with exposure to low doses of radiation. The use of combinations of protective agents has not been extensively tested. Thus, combinations of agents with differing mechanisms of action and/or different toxicities may be superior to single agents in the same manner as combination cancer chemotherapy is often superior to individual agents.

Relative to radiation mitigators, it is an open question as to how long after exposure to radiation the mitigator should still work. Obviously, an agent that works within the first 24 h or longer would be more valuable than an agent that only works within a few hours after exposure, since it may take time to deliver the compound to the site of a nuclear disaster. This would not be a consideration for usage of a protector/mitigator in the radiotherapy clinic, since here the agents can conveniently be given within a few hours of each radiation treatment. An additional consideration applicable to use of a mitigator in civilian or military populations exposed to radiation is that the agent in question should have a convenient mode of administration, e.g., by oral route or by intramuscular or subcutaneous injection. Ideally, the compound should be easily self-administered since access to medical care may be delayed or limited.

Finally, it would be interesting to know if there are other FDA-approved drugs (see section on captopril) or food additives (see sections on DIM and genistein) that exert radioprotective or mitigative activity and could be “repurposed” for these indications.

## Conflict of Interest Statement

The authors declare that the research was conducted in the absence of any commercial or financial relationships that could be construed as a potential conflict of interest.
